# Fibrinogen A Alpha-Chain Amyloidosis in Two Chinese Patients

**DOI:** 10.3389/fmed.2022.869409

**Published:** 2022-04-28

**Authors:** Zhen-Yu Li, Shuang Wang, Dan-Yang Li, Dan Liu, Su-Xia Wang, Xiao-Juan Yu, Gang Liu, Fu-De Zhou, Ming-Hui Zhao

**Affiliations:** ^1^Laboratory of Electron Microscopy, Pathological Center, Peking University First Hospital, Beijing, China; ^2^Renal Division, Department of Medicine, Peking University First Hospital, Beijing, China; ^3^Renal Pathological Center, Institute of Nephrology, Peking University, Beijing, China; ^4^Key Laboratory of Renal Disease, Ministry of Health of China, Beijing, China; ^5^Key Laboratory of CKD Prevention and Treatment, Ministry of Education of China, Beijing, China; ^6^Proteomics Laboratory, Medical and Healthy Analytical Center, Peking University Health Science Center, Beijing, China

**Keywords:** amyloidosis, fibrinogen, LECT2, kidney, gene mutation, mass spectrometry, China

## Abstract

**Objectives:**

Fibrinogen A alpha-chain amyloidosis (AFib amyloidosis) is the most common form of hereditary renal amyloidosis in the United Kingdom and Europe, but has rarely been reported in Asia. In this study, we reported two AFib amyloidosis patients in China, reviewing the literature and summarizing main characteristics of AFib amyloidosis in Asia.

**Methods:**

Two unrelated Chinese patients were diagnosed with AFib amyloidosis by clinical presentation, renal biopsy, mass spectrometry and DNA sequencing in Peking University First Hospital of China from 2014 to 2016.

**Results:**

Both of the patients presented with proteinuria, edema and hypertension. Renal biopsies of two patients showed extensive amyloid deposits (Congo red positive) in glomeruli, and focal tubulointerstitial amyloid deposits was also found in patient 1. Besides, hepatic involvement of amyloidosis has been detected by liver biopsy in patient 1. By electron microscopy, randomly arranged fibrils in a diameter of 8–12 nm was identified in mesangial matrix and subendothelial area of glomeruli. Immunohistochemistry demonstrated amyloid deposits were strongly positive for fibrinogen Aα in glomeruli and positive for LECT2 in the interstitium of renal medulla and the liver in Patient 1. Unevenly positive staining for both fibrinogen Aα and ApoA-I were found in Patient 2. Fibrinogen Aα was the most abundant amyloidogenic protein in both patients identified by laser microdissection and mass spectrometry-based proteomic analysis. Genetic analysis revealed the *fibrinogen A a-chain* gene (*FGA*) mutation in both patients, including a new deletion mutation [c.1639delA (p.Arg547Glyfs^*^21; NM_000508)] in Patient 2. Genetic analysis of the *LECT2* gene in patient 1 revealed a codon change from ATC to GTC at position 172 [c.172A>G (p.Ile58Val; NM_002302)], which is a common polymorphism (SNP rs31517) in all ALECT2 amyloidosis patients.

**Conclusions:**

We reported two AFib amyloidosis patients in China, one of them coexisted with ALECT2 amyloidosis simultaneously.

## Introduction

Amyloidosis is a protein misfolding disorder caused by the deposition of insoluble fibrillar amyloid in extracellular space, resulting in permanent organ damage. It can be either acquired or hereditary. Hereditary amyloidosis comprises a group of disease related to gene mutations in the coding regions of *transthyretin, fibrinogen A*α *chain, apolipoprotein A-I, apolipoprotein A-II, apolipoprotein C-II, apolipoprotein C-III, gelsolin, cystatin C* and *lysozyme*. Among them, transthyretin-derived ATTR amyloidosis presents with polyneuropathy and/or cardiomyopathy, while fibrinogen A alpha-chain amyloidosis (AFib amyloidosis) presents with nephropathy typically.

AFib amyloidosis, which was first described by Benson et al. (1) in a Peruvian-Mexican family, is the most common form of hereditary renal amyloidosis in the United Kingdom and Europe (2), but has been rarely reported in Asia (3). Here we report two AFib amyloidosis patients in China, one of them coexisted with ALECT2 amyloidosis.

## Methods

### Patients

Two unrelated patients presenting with edema and proteinuria were found to have amyloid deposits in kidney: a 29-year-old Chinese woman (Patient 1) and a 47-year-old Chinese man (Patient 2). They underwent detailed clinical examination and laboratory tests including blood and urine biochemistry, electrocardiography, echocardiography and immunofixation electrophoresis of urine and serum.

### Histology and Immunohistochemistry

Renal biopsies from the two patients were examined by routine light microscope (HE, PAS, Masson trichrome, Silver methenamine and Congo red), Immunofluorescence (detection for IgG, IgA, IgM, C3, C1q, FRA, Alb, κ and λ-light chain) and electron microscopy. Immunohistochemistry were performed on formalin-fixed paraffin-embedded renal tissue with antibodies against λ-light chain (1:8000; A0193, Dako), κ-light chain (1:8000; A0191, Dako), fibrinogen Aα chain (1:4000; A0080, Dako, Carpenteria, CA), lysozyme (1:200; GA009929, GTR, Shanghai, China), amyloid A (1:200; M0759, Dako, Glostrup, Denmark), transthyretin (1:2000; A0002, Dako, Carpenteria, CA), gelsolin (1:500; WH0002934M1, Sigma-Aldrich, Germany), apolipoprotein A-I (1:2000; PV9003, Calbiochem) and LECT2 (1:40, AF722, R&D Minneapolis, USA) according to standard techniques. Negative controls were included by omitting primary antibody.

### Laser Microdissection and Mass Spectrometry-Based Proteomic Analysis

Laser microdissection was performed as previously described (4). Briefly, the Congo red-positive areas of renal tissue were microdissected with laser. Proteins extracted from the microdissected areas were digested by typsin, and analyzed by liquid chromatography and electrospray tandem mass spectrometry (LC-MS/MS) to identify the component of amyloidogenic precursors.

### Genetic Investigations

Genomic DNA was extracted from peripheral blood leukocytes as previously described (5). Exon 5 of the *fibrinogen A alpha chain* gene and exon 3 of the *LECT2* gene were amplified by polymerase chain reaction (PCR). Primers were designed using Primer 3.0 online. PCR products were electrophoresed through agarose gel and sequenced with ABI 3730XL (Cangso Medical Inspection, China). The sequences were analyzed using the DNASTAR software. Genomic sequences of the *FGA* gene (NM_000508) and the *LECT2* gene (NM_002302) were used as the reference sequences. Furthermore, online tools such as HGMD Pro, PubMed and 1000Genomes were applied to evaluate the mutation genes.

## Results

### Clinical Findings

Patient 1 was a 29-year-old Chinese woman. She presented with eyelid edema and lower limb edema for 6 months. She was a hepatitis B patient for 14 years and did not receive systemic therapy. Family history was negative. She had been diagnosed as AL amyloidosis in a local hospital 5 months ago and given methylprednisolone and melphalan for 6 days, then stopped due to liver injury. Her blood pressure was 130/70 mmHg when she was admitted into our hospital. The remaining physical examination was unremarkable. Investigations revealed hemoglobin concentration of 96 g/L, proteinuria of 1.74 g per 24 h, serum albumin of 28.8 g/L, serum creatinine of 182.3 μmol/L. Plasma fibrinogen (reference: 1.5–4 g/L) was normal. No evidence of a monoclonal protein was found in serum or urine. No signs of cardiac amyloidosis were found by echocardiography. Liver biopsy revealed amyloid deposition. Eight months after first admission into our hospital, her serum creatinine reached to 608 μmol/L and she started dialysis.

Patient 2 was a 47-year-old Chinese man with 10-month history of intermittent eyelid edema. His mother died of uremia. On admission, his blood pressure was 196/120 mmHg. Physical examination was unremarkable except eyelid edema. He had a nephrotic range proteinuria of 3.6 g per 24 h. Serum albumin was 33.2g/L. His renal function was normal with a serum creatinine of 93.4 μmol/L. Clotting profile was normal. Immunofixation electrophoresis of serum and urine were both negative. Mild thickened intima with a small plaque in the lower extremity artery was found by ultrasound. There was no evidence of heart involvement by echocardiography.

Clinical presentation and laboratory findings of the two patients are summarized in Table 1.

**Table 1:** Clinical presentation and laboratory findings of the patients.

**Patient number**	**Sex/age/family history**	**Clinical presentation**	**Proteinuria(g/24h)/serum albumin(g/L)/serum creatinine(μmol/L)/plasma fibrinogen(g/L)**	**Renal biopsy**	**FGA mutation**
1	Female/29/No	Eyelid and lower limb edema, hypertension	1.74/28.8/182.3/2.58	Amyloid deposits in the glomeruli and interstitium of renal medulla	c.1673delA
2	Male/47/Yes	Eyelid edema, hypertension	3.6/33.2/93.4/2.24	Amyloid deposits exclusively in the glomeruli	c.1639delA

### Histology and Immunohistochemistry (IHC)

**Figure 1:**
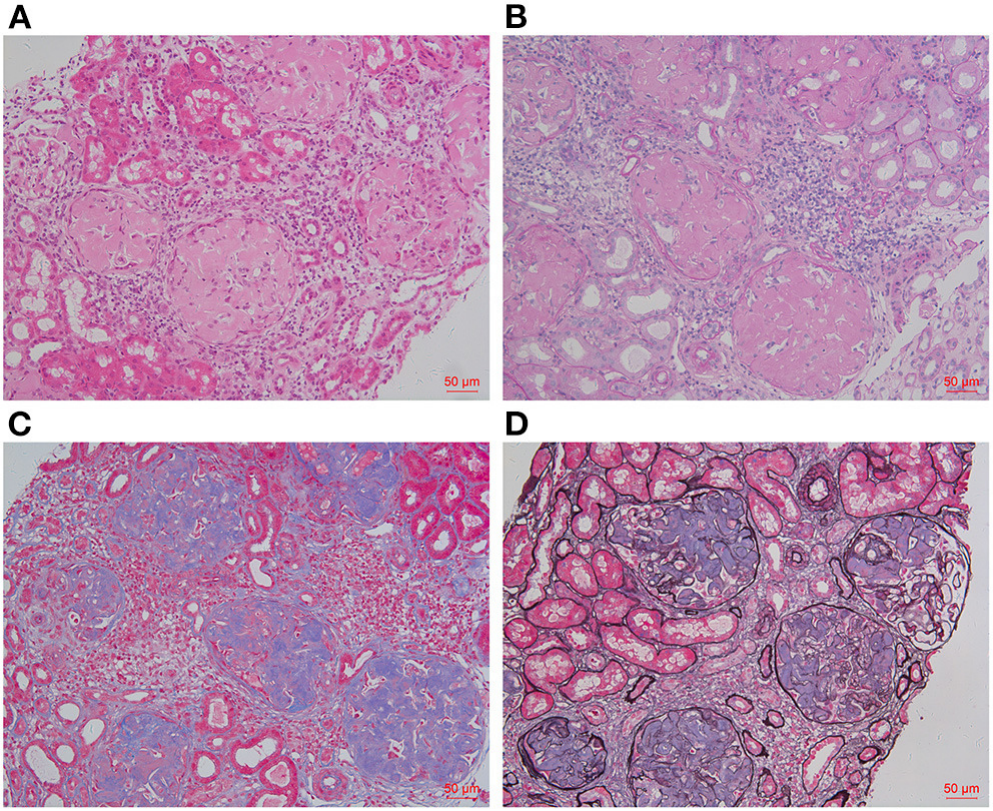
Histology findings of renal biopsy. HE [**(A)**, ×200] and PAS [**(B)**, ×200] staining showed massive amorphous eosinophilic deposits in glomeruli, leading to the obliteration of glomerular capillary loops. These amyloid deposits were pale blue by Masson trichrome staining [**(C)**, ×200], but were not black on Silver methenamine staining [**(D)**, ×200].

**Figure 2:**
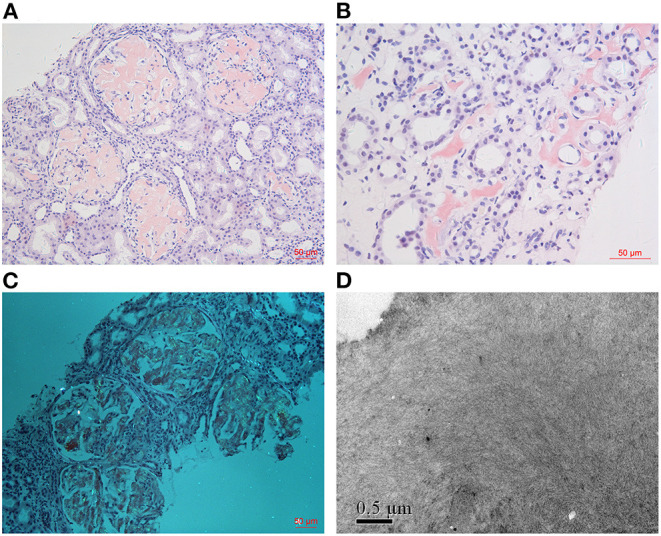
Histology findings (take Patient 1 as an example). Massive homogeneous and Congo red positive deposits were found in glomeruli [**(A)**: Congo red ×200], which produce apple green birefringence under polarized light [**(C)**: Congo red ×200]. Obvious amyloid deposits were found in the renal medulla interstitium of Patient 1 [**(B)**: Congo red ×400]. Electron microscopy showed randomly arranged fibrils in mesangial matrix [**(D)**: EM ×40,000].

Renal biopsies of both patients showed extensive amorphous amyloid deposits (Congo red positive) in glomeruli, resulting in striking glomerular enlargement and almost complete obliteration of the normal architecture (Figures 1A–D, Figure 2A). Congo red positive deposits produced apple green birefringence under polarized light (Figure 2C). No amyloid deposits were found in tubulointerstitium or vessels in Patient 2. But obvious amyloid deposits were found in the interstitium of renal medulla in Patient 1 (Figure 2B). Routine immunofluorescence showed negative staining for light chains (κ and λ). Electron microscope showed randomly arranged fibrils replacing mesangial matrix and subendothelial area (Figure 2D).

**Figure 3:**
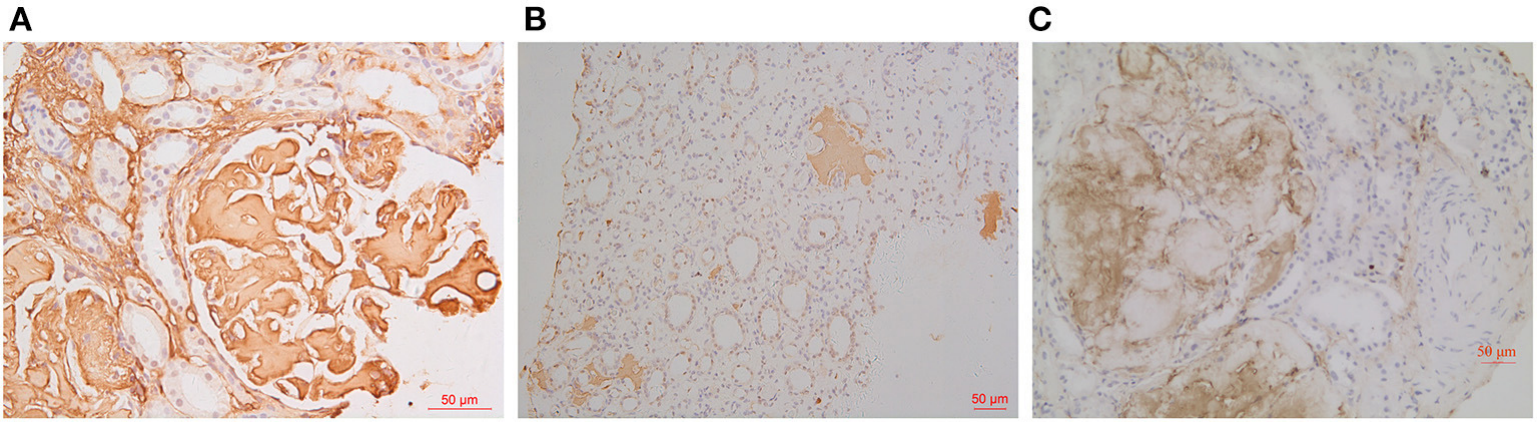
IHC findings. Amyloid deposits were strongly positive for fibrinogen Aα in glomeruli [**(A)**: IHC ×400] and positive for LECT2 in medullary interstitium [**(B)**: IHC ×200] in Patient 1. The glomerular amyloid deposits were unevenly positive for fibrinogen Aα in Patient 2 [**(C)**: IHC ×200].

As for IHC, amyloid deposits were strongly positive for fibrinogen Aα in glomeruli and also positive for LECT2 in medullary interstitium and liver in Patient 1 (Figures 3A,B), and unevenly positive for both apolipoprotein A-I (ApoA-I) and fibrinogen Aα in glomeruli of Patient 2 (Figure 3C). IHC for other amyloidogenic precursors (κ or λ light chains, Amyloid A, transthyretin, lysozyme, gesolin) were all negative.

### Mass Spectrometry-Based Proteomic Analysis

**Figure 4:**
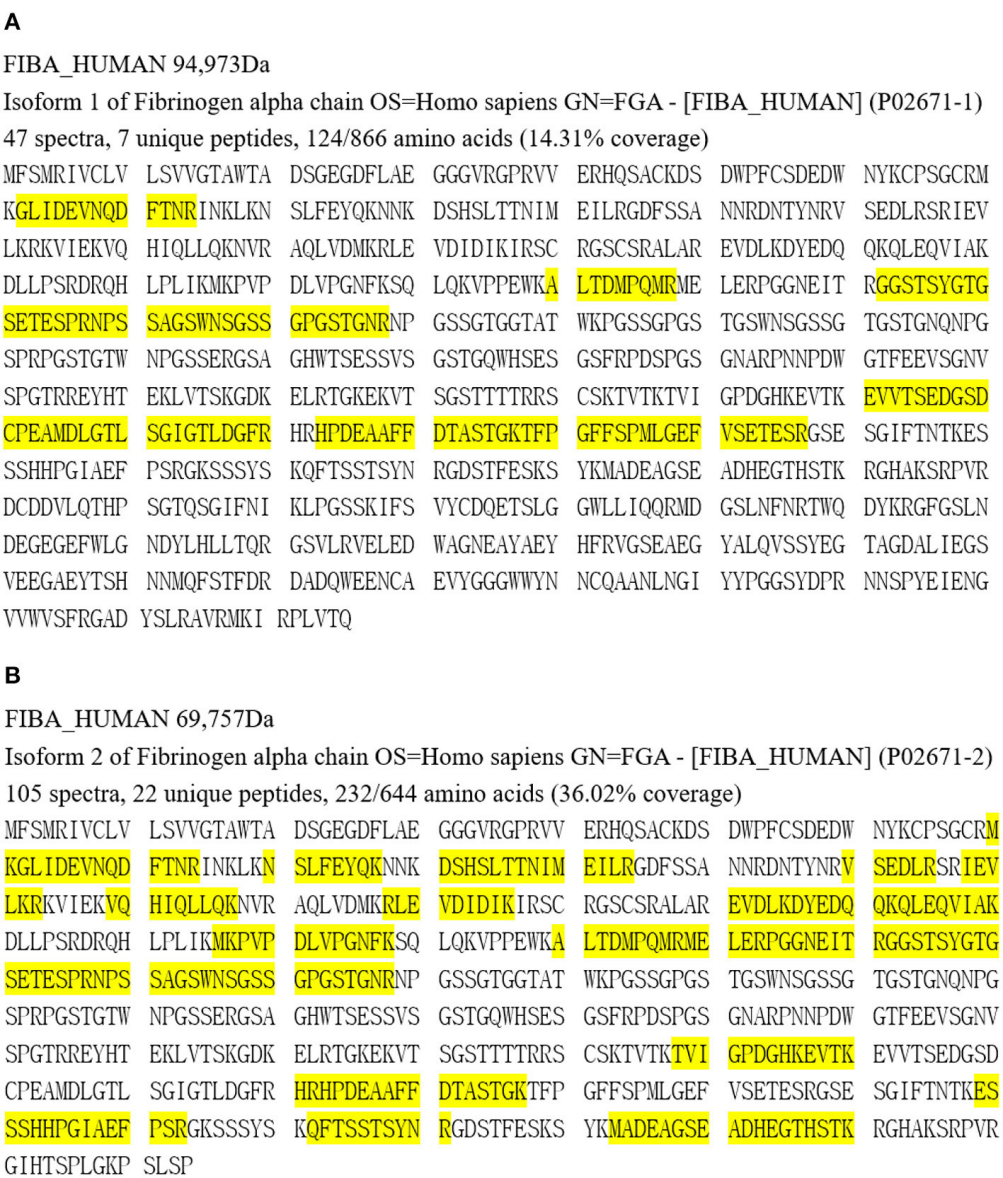
Proteomic analysis results of Patient 1 **(A)** and Patient 2 **(B)**. Yellow part represents covered amino acids.

Apolipoprotein E, serum amyloid P component and apolipoprotein A-IV, which are amyloid signature constituents, were detected in the amyloid deposits of both patients by LC-MS/MS proteomics analysis. Fibrinogen Aα was the most abundant amyloidogenic protein, with 47 spectra, 7 unique peptides for 14.31% coverage in Patient 1 and 105 spectra, 22 unique peptides for 36.02% coverage in Patient 2 (Figure 4).

### Genetic Analysis

**Figure 5:**
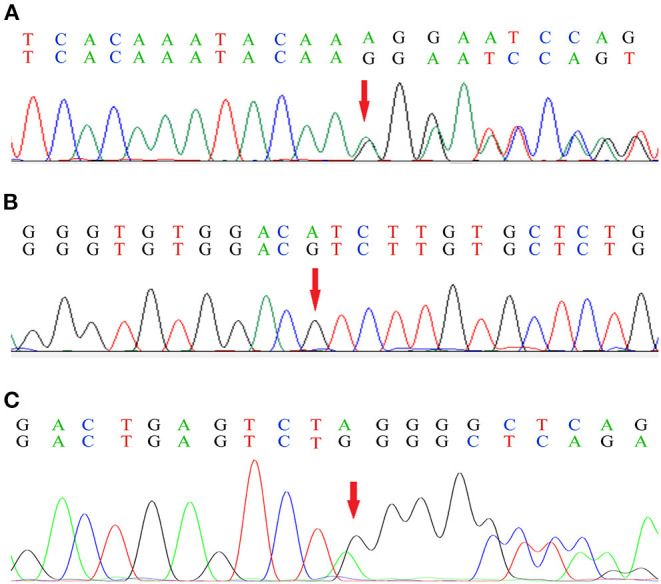
Genetic analysis of the *FGA* **(A)** and the *LECT2* gene **(B)** in Patient 1, and the *FGA* in Patient 2 **(C)**. The base sequence in the first line of each sub-graph is wild type, and the second line is mutant. Red arrow indicates the mutation site.

Genetic analysis of Patient 1 showed a single nucleotide (A) deletion at position 1673 of the *FGA* [c.1673delA (p.Lys558Argfs^*^10), Figure 5A] and a codon change from ATC to GTC at position 172 of the *LECT2* gene [c.172A>G (p.Ile58Val), Figure 5B). In Patient 2, genetic analysis showed a single nucleotide deletion at position 1639 of the *FGA* [c.1639delA (p.Arg547Glyfs^*^21), Figure 5C), resulting in a frame-shift mutation at codon 547.

## Discussion

Fibrinogen is a 340-kD plasma protein produced exclusively by the liver (6) and plays an important role in the coagulation cascade. It is composed of 2 identical heterotrimers, each consisting of 3 polypeptide chains termed Aα, Bβ and γ-chain (7). These polypeptide chains are encoded by distinct genes, *FGA, FGB* and *FGG*. Mutations in these genes are responsible for dysfibrinogenemias, and heterozygous mutations in a small region of C-terminal portion of the Aα chain can cause HRA (8). Wild type fibrinogen Aα chain normally does not result in amyloid deposition (9). AFib amyloidosis is an autosomal-dominant hereditary systemic amyloidosis. To date, 17 amyloidogenic mutations have been described, including 7 missense mutations (c.1627G > A, c.1633G > A, c.1634A > T, c.1670C > A, c.1676A > T, c.1712C > A, c.1718G > T), 8 deletion mutations (c.1611delA, c.1619_1622delTTGT, c.1620delT, c.1622delT, c.1624_1627delAGTG, c.1629delG, c.1632delT, c.1673delA) and 2 insertion-deletion mutations (c.1606_1620del 1619_1620insCA, c.1720_1721delGGinsTT) (3). In our hospital, two patients were diagnosed with AFib amyloidosis, both with deletion mutations.

Unlike Europe, AFib amyloidosis in Asia has been rarely reported (Table 2) and has its own characteristics. The most common variant in Europe is c.1634A > T (E526V) (11), however, there is only one c.1634A > T mutation in the 8 cases of Asia, the other 7 cases are all with novel mutations, include one missense mutation, 4 deletion mutations and one insertion-deletion mutation, making diagnosis more difficult. Same as in Europe, kidney is the predominantly involved organ, which can present with proteinuria, edema, hypertension, and azotemia. But age of onset is significantly earlier (median age, 40 years old) than that in Europe [median age, 58 years old (11)], clinical symptoms seem to be more severe, some patients progressed to ESRD at a younger age. It may be related to more severe genotypes, which were resulted from frameshift mutations. Clinical presentation and gene mutation of AFib amyloidosis patients in Asia are summarized as Table 2.

**Table 2:** Clinical presentation and gene mutation of AFib amyloidosis patients in Asia.

**Patient number**	**Sex/age/family history**	**Ethnicity**	**Clinical presentation**	**FGA mutation**	**Report time**	**References**
1	Female/7/No	Korean	ESRD	c.1606_1620 delATGTTAGGAGAGTTTinsCA	2005	(10)
2	-/40+/Yes	Chinese	-	c.1632delT	2009	(11)
3	-/40+/No	Chinese	-	c.1670C > A	2009	(11)
4	Male/54/No	Chinese	Renal insufficiency	c.1634A > T	2014	(12)
5	Female/32/No	Japanese	ESRD	c.1624_1627delAGTG	2015	(13)
6	Female/33/Yes	Chinese	Renal insufficiency	c.1673delA	2021	(14)
7	Female/29/No	Chinese	Renal insufficiency	c.1673delA	2021	Our case
8	Male/47/Yes	Chinese	Nephropathy	c.1639delA	2021	Our case

*Age, age of onset; ESRD, end-stage renal disease; -, unknown information; 40+, in their fourth decade*.

Diagnosis of AFib amyloidosis is based on clinicopathological findings, immunohistochemistry, mass spectrometry and detection of a *FGA* amyloidogenic genetic variant (15). On renal histology, AFib amyloidosis has its own characteristics with striking glomerular enlargement and exclusive glomerular deposition (11). It is generally recognized that there is little or no vascular or interstitial amyloid deposits in AFib amyloidosis. However, in our study, we found obvious amyloid deposits in the interstitium of renal medulla in Patient 1, which were positive for LECT2 in IHC. Genetic analysis of this patient revealed a single base transversion from A to G at position 172 of the *LECT2* gene [c.172A>G (p.Ile58Val; NM_002302)], this is a common polymorphism (SNP rs31517) that are thought to be present in all ALECT2 amyloidosis patients (16). These evidences support that this patient coexisted with ALECT2 amyloidosis. Moreover, we found that the unevenly staining for amyloid precursors by IHC may lead to a diagnostic pitfall, just like Patient 2 [case 2 has been reported in ref. (13)]. Because proteomic analysis of ApoA-I and genetic analysis of the *ApoA-I* gene were both negative, the unevenly staining of ApoA-I by IHC was thought to be non-specific staining. This makes differential diagnosis of AFib amyloidosis more difficult. IHC is a commonly used method for the identification of amyloid type, however, it fails to identify the amyloid type in up to 30% systemic amyloidosis cases (9). As for our cases, immunohistochemistry was inconclusive in Patient 2. Mass spectrometry-based proteomic analysis is not routinely used in our country due to the high cost, but it can provide reliable information in amyloid type identification. LC-MS/MS proteomic analysis of the two patients corroborated existence of fibrinogen Aα in the amyloid deposits, leading to the diagnosis of AFib amyloidosis finally.

AFib amyloidosis has been rarely reported in China, Patient 1 has been misdiagnosed with AL amyloidosis in a local hospital and given inappropriate chemotherapy. Our experience suggests AFib amyloidosis should also be considered in the differential diagnosis of amyloidosis to avoid inappropriate and even harmful treatments. There is no effective therapy for AFib amyloidosis, treatment is restricted to interrupt further fibril formation, in combination with supportive care of failing organs. For those with ESRD, renal replacement therapy and kidney transplantation can be considered, but amyloid may deposit in the renal graft again. Combined liver and kidney transplantation can prevent the production of amyloidogenic fibrinogen and therefore is curative, but the procedure related mortality is high (17, 18).

## Conclusion

Here we report two AFib amyloidosis patients in China, one of them coexisted with ALECT2 amyloidosis. Patients with AFib amyloidosis do exist in Asia, most of them had earlier onset age, with novel frameshift mutations. AFib amyloidosis may coexist with other types of amyloidosis, physicians should pay more attention to the differential diagnosis to avoid inappropriate and even harmful therapy.

## Ethics Statement

Written informed consent was obtained from the individual(s) for the publication of any potentially identifiable images or data included in this article.

## Author Contributions

Z-YL, SW, and D-YL performed the experiments of IHC, pathological studies, and gene testing. DL performed the mass spectrometry. X-JY, GL, F-DZ, and M-HZ was in charge of patient care and clinical data collection. Z-YL drafted this manuscript. S-XW designed this study and revised the manuscript critically for important intellectual content. All authors read and approved the final manuscript.

## Conflict of Interest

The authors declare that the research was conducted in the absence of any commercial or financial relationships that could be construed as a potential conflict of interest.

## Publisher's Note

All claims expressed in this article are solely those of the authors and do not necessarily represent those of their affiliated organizations, or those of the publisher, the editors and the reviewers. Any product that may be evaluated in this article, or claim that may be made by its manufacturer, is not guaranteed or endorsed by the publisher.
